# The Impact of Misinformation on Social Media in the Context of Natural Disasters: Narrative Review

**DOI:** 10.2196/70413

**Published:** 2025-07-31

**Authors:** Sonya Hilberts, Mark Govers, Elena Petelos, Silvia Evers

**Affiliations:** 1 Department Health Services Research, CAPHRI School at Faculty of Health, Medicine and Life Sciences, Maastricht University, Bonnefantenstraat 2, Maastricht, 6211 KL, The Netherlands, 61455420982; 2 Clinic of Social and Family Medicine, Faculty of Medicine, University of Crete, Heraklion, Greece

**Keywords:** misinformation, infodemic, social media, natural disaster, preparedness

## Abstract

**Background:**

Misinformation on social media during natural disasters has become a significant challenge, with the potential to increase public confusion, panic, and distrust. Although individuals rely on social media platforms for timely updates during crises, these platforms also facilitate the rapid spread of unverified and misleading information. Consequently, misinformation can hamper emergency response efforts, misdirect resources, and distort public perception of the disaster’s true severity.

**Objective:**

This narrative review aims to (1) critically evaluate the available evidence; (2) unpack the dynamics of misinformation on social media in the context of natural disasters, specifically natural hazards, shedding light on the challenges, implications, and potential solutions; and (3) develop a conceptual model linking misinformation, public impact, and disasters, grounded in sourced evidence.

**Methods:**

The narrative review examines the impact of social media misinformation in the context of natural disasters. The literature search was conducted using the PubMed database and Google Scholar in April 2024. Studies eligible for inclusion were published in English, with no restrictions on publication date, geographic region, or target population. The inclusion criteria focused on the original research that examined social media misinformation related to natural disasters, specifically natural hazards.

**Results:**

From an initial pool of 173 studies, 9 studies met the inclusion criteria for this review. The selected studies revealed consistent patterns in how misinformation spreads during natural disasters, highlighting the role of users, some influencers, and bots in amplified false narratives. The misleading messages disseminated across social media platforms often outpaced official communications, resulting in reduced trust and exacerbating anxiety, stress, and fear among affected populations. This heightened emotional response and erosion of trust in official communications influenced an individual’s susceptibility to the misinformation and prompted inappropriate actions. Consequently, such actions led to resource misallocation, overwhelmed emergency services, and diverted attention away from genuine needs. Collectively, these factors negatively impacted public health outcomes and diminished the effectiveness of emergency management efforts, as illustrated in the conceptual model developed to provide a greater understanding of this critical area of study.

**Conclusions:**

This narrative review highlights the significant impact of misinformation in the context of natural disasters, specifically natural hazards. It stresses the urgent need for disaster preparedness and response plans that include targeted interventions such as real-time misinformation detection technologies, public education campaigns focused on digital literacy, and proactive debunking initiatives. Implementing these strategies can help mitigate the harmful effects of misinformation, strengthen public trust in official communications, enhance the effectiveness of disaster response, and improve public health outcomes.

## Introduction

Social media platforms have increasingly become essential tools during natural disasters, enabling real-time communication, rapid information dissemination, and enhanced public awareness and safety [1]. However, these platforms also facilitate the rapid spread of misinformation, significantly complicating disaster management and public health responses [1-3]. The dissemination of misinformation during disasters can lead to heightened public anxiety, confusion, resource misallocation, reduced effectiveness of emergency responses, and diminished trust in official communications [4-7]. For example, during Hurricane Sandy in 2012, false tweets about the New York Stock Exchange flooding and fabricated images of the storm spread widely, causing public panic and confusion [4]. Similarly, misinformation during Hurricanes Harvey and Irma in 2017 led to widespread fears about mandatory ID checks at shelters, discouraging undocumented immigrants from seeking safety [5]. Furthermore, the spread of misinformation can also delay emergency response efforts, as observed in the 2018 Kerala floods, where misleading social media posts hindered rescue operations [6].

A key challenge in addressing misinformation during natural disasters lies in the timing and immediacy of such events. They often occur with little warning, leaving minimal time for preparation. For example, while a hurricane may be predicted a few days in advance, its impact is typically sudden and severe. This rapid onset and escalation of natural disasters can amplify the spread of misinformation on social media platforms in unique ways. For this reason, examining misinformation specifically within the context of natural disasters offers valuable insights into the role of social media in crisis communication.

Additionally, the sheer volume of misinformation circulating online poses substantial challenges for government agencies, humanitarian organizations, and health systems, entities that are paradoxically central to disaster response. To counter these challenges, coordinated efforts between governments, social media platforms, and the public are essential to ensure the integrity of information during disasters. For example, automated misinformation detection systems implemented by platforms like Twitter (subsequently rebranded X) have been proven beneficial during hurricanes by flagging misleading content related to emergency shelters and procedures [5]. In parallel, public digital literacy initiatives have demonstrated potential in reducing misinformation by the public’s ability to critically assess online content [8]. Nevertheless, research suggests that misinformation spreads more rapidly when official communications are unclear or delayed, highlighting the need for timely, transparent, and proactive crisis communication strategies [9].

There are several terms to describe inaccurate information, such as fake news, rumors, propaganda, infodemic, disinformation, and misinformation. This review will focus on misinformation, while adopting a broader lens to also encompass false information intentionally shared (disinformation), as it provides a more holistic account of the impact on public health and disaster response, whether intentional or not.

While numerous accounts document specific incidents of misinformation during events such as hurricanes, floods, and earthquakes [4-6,9], much of the literature focuses on isolated cases. This narrow focus limits our understanding of broader patterns and systematic impacts across different types of disasters and geographic regions. The absence of an integrated synthesis constrains our ability to comprehend how misinformation influences public perceptions, behavioral responses, and the overall effectiveness of emergency response [2,10,11].

Therefore, this narrative review aims to address this gap by synthesizing and consolidating available evidence on misinformation on social media during natural disasters, identifying key patterns, dynamics, and impacts. Additionally, a conceptual model will be developed as part of the review to clarify the relationships between misinformation origins, public impact, and disruptions in emergency responses, informed by sourced evidence [4-6,8,9,12-15]. By synthesizing available research, this review may support policy makers, emergency responders, and public health officials in designing targeted strategies to mitigate the spread of misinformation. Ultimately, strengthening the resilience of disaster response systems and safeguarding public health outcomes.

## Methods

### Study Design

The methodology for this narrative review is grounded in established principles from relevant literature to guide the review process effectively. These encompass a structured process and have been adapted from the guidelines recommended by the Joanna Briggs Institute [16] as follows.

- The topic and research question were defined, and the criteria that determine whether literature will be included or excluded were created.
- A search strategy was developed and executed, specifying keywords, subject headings, and Boolean operators.
- An initial screening of titles and abstracts from the search results was conducted to assess relevance.
- A detailed full-text review of articles was performed, based on predefined inclusion and exclusion criteria. The screening process results were documented using a PRISMA (Preferred Reporting Items for Systematic Reviews and Meta-Analyses) flowchart for transparency.
- Relevant information was extracted from the included studies to support and inform the findings of the review using a standardized data extraction tool.

Also, the Cochrane Handbook for Systematic Reviews of Interventions was used to develop the protocol for this review (PROSPERO [International Prospective Register of Systematic Reviews] CRD42024542111).

The initial search was done by SH. The screening and the full text review were done by SH and EP. Data extraction and synthesis were done by all authors. Any disagreement was solved in consensus meetings, which led to an agreement. The research team consisted of members with different backgrounds relevant to this study.

SH’s experience supporting disaster management, digital transformation programs, and national COVID-19 pandemic response initiatives has deeply influenced her approach toward organizational efficiency, resilience, and strategic foresight.

MG, an associate professor of the organization, management, and digital, applies sociotechnical systems thinking to align social and technical dimensions in health care and business, advancing theory by linking STS principles to digital transformation challenges while also integrating broader cultural, behavioral, and political perspectives on social phenomena.

EP is a public-health specialist and senior lecturer (University of Crete, University of West Attica, and Maastricht University) whose 2-decade career spans R&D (Research and Development), evidence generation and synthesis, HTA, digital health, and artificial intelligence (AI). Since returning to academia, she combines academic research with policymaking and advocacy work, currently serving as president for Global Health and vice president for HTA (Health Technology Assessment) at The European Public Health Association (EUPHA) and chair for RWE & AI at HTAi.

SE, Professor of Public Health Technology Assessment and Scientific Director of the Care and Public Health Research Institute, specializes in health-economic review methods, scoping, narrative, systematic, and meta-analyses and created the widely adopted Consensus Health Economic Criteria list and accompanying guidelines for systematic reviews of economic evaluations.

All research team members are aware that experiences derived from their different roles and positions have shaped their own perspectives. However, the diversity in backgrounds helped to broadly reflect on the findings of this study.

#### Search Strategy

The search was conducted in April 2024. The primary source to retrieve information was the PubMed database, because of its primacy as a reputable source of public health research. The paucity of results from that source led to expanding the search, adding free searching on Google Scholar, for works published in the English language.

The decision to limit the selection to English-language papers was based on 3 factors. First, the research team possesses proficiency in English, which ensures accurate interpretation and analysis of the included studies. Second, the databases used for literature searches (eg, PubMed) primarily index journals in the English language. Finally, conducting a multilingual review would require significant resources, including translation and verification by native speakers of each language.

There was no date restriction imposed to ensure the capture of all relevant publications related to misinformation on social media in the context of a natural disaster, specifically related to natural hazards. Also, no restriction was imposed on the country or on the age of the target populations.

The search process began with the identification of relevant keywords or phrases that would deliver the desired review results.

A simple search was then done using free text terms which included a search on the topic, looking at words in subject headings, titles, abstracts, and authors’ keywords, also scanning for synonyms, alternative spelling variants, acronyms, abbreviations, encompassing (1) “exploded” subject heading, include narrower subject headings found in the hierarchy as free text terms; (2) text mining (subject terms, index terms, descriptors, and MeSH [Medical Subject Headings]); (3) truncation was used where a search for a term that begins with a word was needed; and (4) once all free-text terms and controlled vocabulary terms had been identified, the next step was to use the correct Boolean operators to combine the terms using “or” OR “and.”

The three categories of interest were (1) “misinformation” (also “infodemic”), (2) “social media,” and (3) “natural disaster” (expanded to include 4 specific types: earthquake, fire, flooding, and tsunami). Combinations of these 3 categories of terms were applied to search titles, abstracts, subject headings, and author keywords. The search strategy also included synonyms, alternative spellings, acronyms, and abbreviations to ensure inclusive coverage of the topic. The review then used the snowballing technique to identify additional studies cited from the studies retrieved.

Peer-reviewed research reports published in journals and conference proceedings were eligible for inclusion. There was no restriction on the date published or the type of original research studies. This included research regarding natural field experiments, observational analysis, surveys, samples, and public information gathered through web scraping and social mining techniques. Systematic reviews meeting the eligibility criteria were examined for additional relevant references.

#### Inclusion Criteria

Studies were included if the authors only addressed misinformation on social media in the context of a specific instance of a natural disaster.

There was no restriction on the date published or the type of original research studies. This included research regarding natural field experiments, observational analysis, surveys, samples, and public information gathered through web scraping and social mining techniques.

#### Exclusion Criteria

The studies that did not investigate misinformation on social media in the context of a natural disaster or that were not in English, and duplicates of another paper, were also removed after screening. Also, studies that solely focused on how to design and build tools to detect misinformation were also excluded.

In addition, the exclusion criteria comprised editorials, letters to the editor, systematic reviews, abstracts, protocols, workshop summaries, perspectives, opinions, diagnosis methods, books, and book chapters, as well as summaries of other reviews, which were excluded before further screening. A documented record was kept of the search findings and translated into a PRISMA flow diagram [17] to provide transparent and complete reporting.

#### Data Extraction (Selection)

We used Microsoft Excel software to capture and synthesize the data. For the included studies, the elements in Tables S2 and S3 found in Multimedia Appendix 1 were analyzed in the full text. The characteristics of the included studies were assessed using a predefined criterion.

#### Study Characteristics

Each study was analyzed for the demographics, context, geographical location, study methods, objectives, study setting, data analysis techniques, the topic of interest, the period of time, data sources, data size, the collecting data method, misinformation effects, and lessons learned.

#### Evidence Synthesis

Evidence synthesis includes the type of social media platform, type of natural disaster, type of misinformation, public impact, and who is impacted.

The aim of the study is to (1) critically analyze the available evidence; (2) explore the dynamics of misinformation on social media in the context of natural disasters, shedding light on the challenges, implications, and potential solutions in this critical domain; and (3) the development of a conceptual model linking misinformation, public impact, and natural disasters based on available evidence from the papers sourced.

For the purpose of this review, the term “natural disaster” is used to explicitly refer to natural hazards, including but not limited to hurricanes, earthquakes, floods, tornadoes, and wildfires. The focus on natural hazards allows for a targeted exploration of how misinformation spreads and impacts populations during these specific types of crises.

Also, the conceptual model is obtained from the 9 studies to gain a greater understanding when examining the relationship between misinformation on social media in the context of natural disasters.

#### Definition of Terms

A disaster, according to the United Nations Office for Disaster Risk Reduction, is: “A serious disruption of the functioning of a community or a society at any scale due to hazardous events interacting with conditions of exposure, vulnerability, and capacity, leading to one or more of the following: human, material, economic, and environmental losses and impacts” [7]. Disasters can be caused by various kinds of hazards [18,19] and can have devastating impacts on people and communities. Disasters linked to natural hazards, including widespread fires, floods, storms, earthquakes, and droughts, may result in significant damage and loss of lives.

Disinformation is commonly defined as false information intentionally shared to deceive [20,21]. 

Infodemic, which was originally coined by David J Rothkopf in 2003, describes the overabundance of information, including misinformation, associated with significant events such as a federal election, pandemics, or natural disasters [22,23]. 

Misinformation is defined as the dissemination of inaccurate information without the intention to deceive [20,21].

Public health is the science and practice of preventing disease, extending life expectancy, and promoting overall health through organized societal efforts [24].

Public impact is defined as the influence or effect that actions and events have on the public [25].

Social media is defined as “a group of Internet-based applications that build on the ideological and technological foundations of Web 2.0, allowing the creation and exchange of user-generated content” [26].

## Results

### Overview

**Figure 1.**
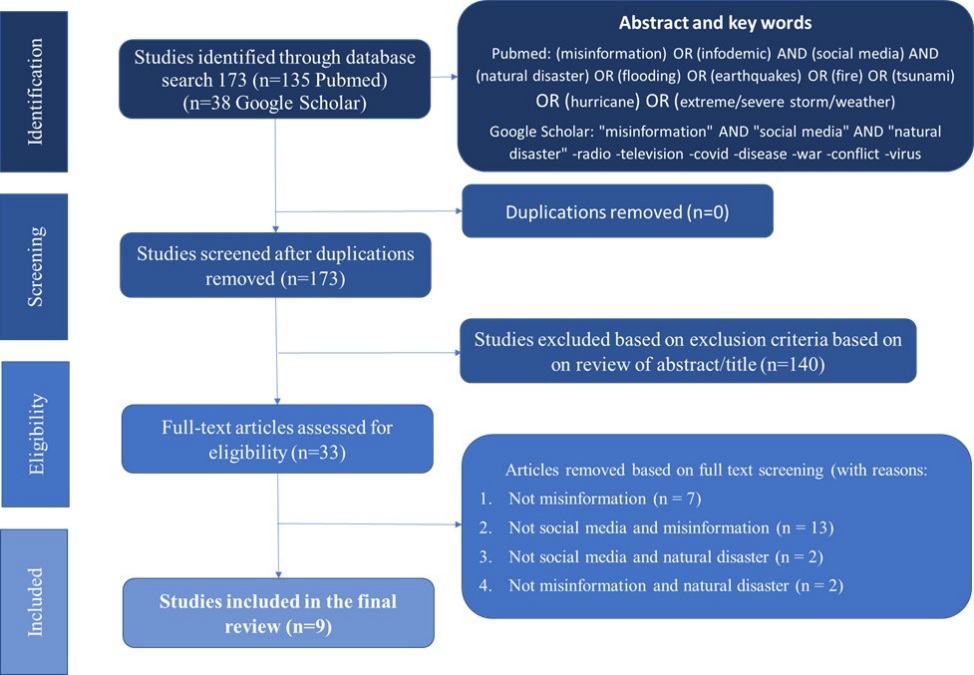
PRISMA (Preferred Reporting Items for Systematic Reviews and Meta-Analyses) flow diagram of the search findings.

Initially, 173 studies were screened for eligibility based on their titles and abstracts, reducing the number of potential studies to 33. Following a full-text review, 9 studies met the inclusion criteria and were included in this narrative review (Figure 1).

Table 1 summarizes the key characteristics of the 9 studies selected for the review. Of these, 4 studies used a case study design, 4 studies used content analysis, and 1 study used survey methods. All studies analyzed data extracted from social media platforms; 8 studies focused on Twitter, while 1 study examined several social media platforms.

**Table 1.** Summary of findings of characteristics of the 9 studies.

Author	Country of origin	Type of study and source	Social media platform	Type of natural hazard (disaster)	Type of misinformation	Study size
Gupta et al (2013) [4][Bibr R4]	United States	Content analysis, peer-reviewed conference paper	Twitter (subsequently rebranded X)	Hurricane Sandy	Fake images of sharks swimming in the streets and manipulated photos of storm damage.	1.8 million tweets from 1.2 million unique users, with 10,350 tweets containing fake images and 5767 tweets containing real images.
Hunt et al (2020) [5]	United States	Case study, peer-reviewed	Twitter (X)	Hurricanes Harvey and Irma.	False claims that immigration status checks were being conducted at evacuation sites and shelters.	Hurricane Harvey: 2032 unique tweets (1440 debunking tweets). Hurricane Irma: 601 unique tweets (259 debunking tweets).
Vasudevan and Alathur (2022) [6][Bibr R6]	India	Case study, peer-reviewed	WhatsApp and Facebook	Heavy flooding	Misleading information on emergency instructions.	561 completed a survey who were affected by the flood in Kerala.
Rajdev and Lee (2015) [8][Bibr R8]	United States	Thesis: case study, peer-reviewed conference paper	Twitter (X)	Moore Tornado and Hurricane Sandy	Spam and fake messages.	Collected 1% sample tweets posted during a period of each of the 2 natural disaster events.
Zhai et al (2023) [9]	United States	Case study, peer-reviewed	Twitter (X)	Hurricane Sandy	False information about the disaster’s impact and situation, such as exaggerated damages or incorrect emergency instructions.	691 tweets.
King and Wang (2023) [12]	United States	Content analysis, peer-reviewed	Twitter (X)	Hurricane Harvey	Misleading information leading to changes in perception.	42 million tweets with 3589 original verified real or false tweets cross-checked with fact-checking websites and relevant federal agencies.
Dallo et al (2023) [13]	Global	Content analysis peer-reviewed	Twitter (X)	Earthquake	About the ability to predict earthquakes.	82,129 tweets.
Oh et al (2010) [14][Bibr R14]	Haiti	Content analysis, peer-reviewed conference paper	Twitter (X)	Haiti Earthquake	False claims about aid offers.	962 tweets.
Abdullah et al (2015) [15]	Japan	Survey peer-reviewed	Twitter (X)	Generically focused	Unverified information and rumors.	133 participants (students from Iwate Prefectural University, Japan).

The narrative review evaluated the available evidence on misinformation via social media in the context of natural disasters and developed a conceptual model to enhance understanding of the intersection of misinformation origin, public impact, and emergency response disruption during natural disasters. The term “natural disaster” is used explicitly to refer to natural hazards, including but not limited to hurricanes, earthquakes, floods, tornadoes, and wildfires. Focusing on natural hazards enabled a targeted examination of how misinformation spreads and affects populations during these specific types of crises.

#### Critical Analysis of Available Evidence

The 9 studies reviewed provide powerful insights into how misinformation spreads and the impacts to communities during natural disasters. Synthesizing evidence across key criteria, such as misinformation type and the social media platforms used, reveals distinct challenges posed by different disaster contexts (Multimedia Appendix 1) [4-6,8,9,12,14,15].

#### Type of Misinformation and Natural Disasters

Recurring themes in disaster-related misinformation include exaggerated reports, misleading emergency instructions, false predictions, and inaccurate information concerning public safety measures. Such misinformation not only disrupts disaster response efforts but also disproportionately affects vulnerable populations. MultimediaAppendices 2  3 illustrate the range of disasters examined and the corresponding misinformation trends identified in each case, highlighting the pervasive and far-reaching consequences of misinformation during crises.

Four studies focused specifically on hurricanes [4,5,9,12] and one on Hurricane Sandy and the 2013 Moore Tornado [8]. Two studies focused on earthquakes [13,14], 1 study was generically focused on “disasters” [15], and 1 study focused on the Kerala floods [6].

#### Social Media Platforms and the Spread of Misinformation

The reviewed studies indicate that misinformation during disasters spreads primarily through popular social media platforms, particularly Twitter (X), Facebook, and WhatsApp.

Twitter was identified by 8 studies as a social media platform for the dissemination of misinformation [4,5,8,9,12-15]. These studies emphasized Twitter’s role in spreading rumors, false predictions, and misleading information during disasters such as Hurricane Sandy, Hurricanes Harvey and Irma, the 2013 Moore Tornado, and the Haiti and Tohoku earthquakes.

Social media platforms like Facebook, WhatsApp, and Twitter are essential for rapid disaster communication, enabling institutions to disseminate critical updates, supporting evacuation efforts, and facilitating resource allocation such as food and medical supplies [6]. For instance, during Hurricane Sandy, social media was crucial for disseminating safety updates [9]; however, these platforms also acted as conduits for misinformation.

The dual role of social media, as both a valuable communication tool and a channel for misinformation, highlights its complex influence on public perception and response efforts [15]. An example of this was seen during the 2018 floods in Kerala, India, where WhatsApp served as the main communication channel for identifying places of safety and communicating the needs of affected individuals. However, this same communication channel was exploited for spreading misinformation, causing confusion and disruption to relief operations [6]. The rapid and unverified dissemination of false information among affected communities contributed to the chaos in relief operations.

The duality inherent in social media highlights the importance of actively monitoring and managing these platforms to ensure they aid rather than hinder disaster response efforts [5].

#### Impact of Exposure to Misinformation in the Context of a Natural Disaster

Misinformation during natural disasters can profoundly affect the public and disrupt disaster response systems. Vulnerable populations, along with the general public, face heightened risks as misinformation spreads through various sources, including individuals, influencers, bots, and fake profiles, leading to widespread confusion and panic [14,15].

The 9 studies reviewed highlight the rapid and far-reaching consequences of misinformation. Several studies reported associations between misinformation circulating on social media and heightened public panic, perceived misallocation of resources, and a decline in trust in emergency response systems [14]. Public confusion and fear are particularly acute when misinformation distorts the perceived severity of a disaster or spreads false emergency instructions [4,6,8,9,12,14,15].

For example, during Hurricane Sandy, exaggerated reports of damage and incorrect emergency instructions were circulated on Twitter, leading to public confusion and resource misallocation [8]. Similarly, false predictions about earthquakes shared on Twitter triggered panic and disrupted effective response strategies [13-15]. Furthermore, during the Kerala floods, WhatsApp emerged as a major source of misinformation, where false reports about aid and evacuation points delayed relief efforts [6].

In 1 notable instance, misinformation was specifically targeted at immigrant communities. During Hurricanes Harvey and Irma, false claims about immigration enforcement at evacuation sites generated fear and deterred individuals from seeking help [5].

Exposure to conflicting reports and unverified claims makes it challenging for the public to discern credible information, leading to skepticism and diminished trust in authorities and media sources [4].

Multimedia Appendix 4 presents the 3 key themes identified from the 9 studies illustrating the public impact from misinformation during a natural disaster, emphasizing its disruptive effects on trust, resource allocation, and public safety during natural disasters.

The evidence highlights the critical need for robust strategies to combat the spread of misinformation during natural disasters. Suggested approaches include digital literacy campaigns, timely debunking efforts, and coordinated action between governments, social media platforms, and public health organizations. Addressing these challenges is essential for strengthening emergency response systems and restoring public trust in official information.

### Dynamics of Misinformation on Social Media in the Context of Natural Disasters

The dynamics of misinformation on social media during natural disasters represent a complex phenomenon that poses substantial challenges while also offering opportunities for targeted interventions. The rapid and widespread dissemination of misinformation endangers public safety and disrupts response efforts, yet it simultaneously highlights the potential for developing tailored mitigation strategies.

In this context, dynamics refers to the evolving interplay between social behaviors, technological mechanisms, and emotional responses that influence how misinformation originates, spreads, and influences public understanding and disaster response. An understanding of these dynamics, including the associated challenges, implications, and potential responses, is essential for designing effective strategies to mitigate the impact of misinformation and improve disaster resilience.

#### Challenges

The role of AI in misinformation management is inherently bidirectional. On one hand, algorithmic tools can detect and counter false information, while on the other hand, social media content algorithms often prioritize engagement over accuracy, curating personalized news feeds that amplify sensational content. As a result, misinformation, particularly that which evokes strong emotional responses, often receives greater visibility than verified information [5]. Platforms such as Twitter, Facebook, and WhatsApp facilitate the rapid sharing of sensational or emotionally charged, easier-to-read content and tend to resonate more with users experiencing stress during disasters. The emotional resonance contributes to the accelerated spread of misinformation [4]. The speed and volume of such content can overwhelm emergency communication channels, confuse the public, and erode trust in official sources [5,8,9,14].

Furthermore, the decentralized nature of social media allows anyone to act as a source of information, regardless of credibility. This absence of gatekeeping makes it difficult for the public to distinguish between trustworthy sources and those spreading misinformation. The resulting confusion can lead to panic, as evidenced during Hurricane Sandy and the Kerala floods, where false reports led to delayed relief efforts [4,6,8].

The influencers, users with a substantial social media following, exacerbate the problem. During the 2013 Moore Tornado, high-profile accounts were responsible for the spread of misinformation, which misdirected resources and heightened public confusion [8].

Compounding these structural issues is the psychological environment of disaster contexts. Zhai et al [9] observed that emotionally charged misinformation is more likely to resonate with fearful and uncertain audiences. This resonance increases the likelihood of misinformation being shared, creating a feedback loop in which it spreads rapidly and becomes increasingly more difficult to debunk.

These dynamics highlight the complexity of misinformation during disasters and the critical importance of regulating influential content sources, strengthening digital literacy, and enhancing platform accountability.

#### Implications

The consequences of misinformation during natural disasters are immediate and far-reaching. It not only causes immediate confusion and panic but also delays emergency responses and undermines long-term trust in official information sources. These effects persist well beyond the disaster itself. When the public is exposed to conflicting or inaccurate information during a disaster, their trust in future communications is diminished, making it more difficult for authorities to manage subsequent emergencies effectively [13,27].

As noted by Dallo et al [13], this erosion of trust weakens community cohesion and reduces collective disaster resilience. When public trust deteriorates, communities are less able to respond in a unified and effective manner, potentially leading to greater societal fragmentation and reduced capacity to withstand future disasters [28]. These outcomes highlight the importance of sustained efforts to build and maintain public trust in official communications during disasters.

Additionally, misinformation released during disasters can lead to misallocation of resources, inappropriate public behavior, and delayed emergency response. When individuals act on misinformation, such as evacuating in response to nonexistent threats or requests for aid in areas not requiring immediate need, emergency services can become overwhelmed [4,6,12]. For instance, Rajdev and Lee [8] reported how misinformation during Hurricane Sandy misled the public into taking misguided actions, which strained emergency resources and complicated the overall response effort. In such cases, disaster management teams must contend not only with the crisis but also with the secondary challenges created by misinformation.

#### Potential Solutions

Addressing the challenges posed by misinformation during natural disasters requires a multipronged approach. One key solution involves the use of advanced technological tools, such as AI algorithms, to detect and flag misinformation on social media platforms. These systems can identify patterns of misinformation and alert both users, official agencies, and platform administrators, enabling timely corrective action [5,9,14]. Additionally, partnerships with fact-checking organizations allow social media platforms, social media platforms to implement real-time verification mechanisms, which can help curb the rapid spread of unverified content [14].

Public education also plays a crucial role in countering misinformation. Digital literacy programs that teach individuals how to assess the credibility of online content can reduce susceptibility to misinformation during disasters. Such programs should emphasize critical thinking and promote awareness of the risks associated with sharing unverified information [8,14,29]. Furthermore, targeted educational campaigns aimed at specific demographics, such as older adults, who are more likely to unknowingly spread misinformation, can be particularly effective [13].

Moreover, collaboration between governments, social media platforms, and public health institutions is essential. Through strategic partnerships, these entities can coordinate the dissemination of timely, verified information during disasters [4,6]. Such collaborations should prioritize amplifying credible sources and adjusting platform algorithms to promote accurate information over sensationalized or misleading content. Furthermore, proactive engagement such as preemptive debunking and real-time updates during a disaster can help to limit the spread and influence of misinformation [5,9,14,30].

Communication strategies must also account for the emotional responses that misinformation can trigger during disasters. Understanding the emotional dynamics within affected communities enables the development of messages that are empathetic, clear, and trustworthy. This approach can help reduce panic and confusion while reinforcing public trust in official sources. For example, during the Kerala floods, misinformation shared via WhatsApp contributed to widespread anxiety and disrupted relief efforts [6]. Emotionally sensitive communication may alleviate distress and enhance the overall effectiveness of disaster response.

Finally, predisaster preparedness initiatives should include public education about misinformation and promote critical thinking before a natural disaster occurs. Programs such as community disaster preparedness events can inform the public about the dangers of misinformation and encourage reliance on official communication channels [13]. The proactive awareness campaigns are vital for ensuring that individuals are better equipped to navigate the complex information landscape during disasters, thereby strengthening societal resilience and reducing the harmful impact of misinformation.

### Conceptual Model Linking Misinformation, Natural Disasters, and Public Impact

**Figure 2.**
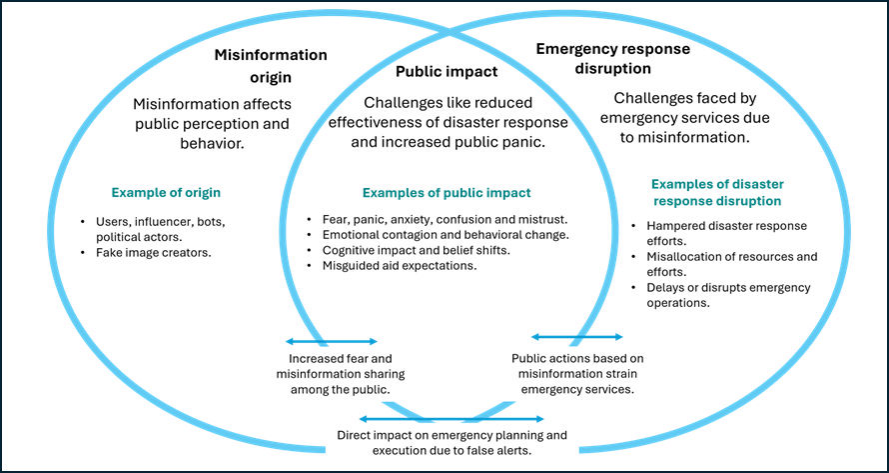
Conceptual model extrapolated from the 9 studies, illustrating how misinformation on social media impacts the public and disrupts disaster recovery.

The research findings underscore the multifaceted impact of misinformation on natural disasters and the public. The conceptual model presented in Figure 2 [4-6,8,9,12,14,15], structured as a Venn diagram, offers a systematic framework that has been largely absent in existing literature. It elucidates the complex interrelationships between the origins of misinformation, its effects on public perception, and the resulting disruptions to emergency response systems during disasters.

The conceptual model synthesizes findings from the 9 included studies, organized into 3 interrelated domains: misinformation origin, public impact, and emergency response disruption. Each domain was examined through extraction and thematic analysis of study-level data, enabling the identification of key patterns related to misinformation actors, societal reactions, and institutional challenges (Figure 2).

By illustrating the intersections and overlaps between the domains, the model highlights the compounding effects of misinformation in disaster contexts, demonstrating how these dynamics escalate challenges and increase the burden on response systems [1].

The interconnected nature of these concepts suggests that efforts to identify and address misinformation at its source may enhance public health outcomes and improve disaster response effectiveness.

While misinformation is typically unintentional, in the context of natural disasters, it may be amplified by coordinated disinformation campaigns, automated bots, or actors with strategic intent. This convergence increasingly blurs the distinction between misinformation and disinformation.

#### Misinformation Origin

Misinformation during emergencies often originates from a range of sources, including ordinary users, social media influencers, bots, and fake accounts [4,5,13-15]. Ordinary users may unknowingly share unverified or manipulated content, often motivated by a sense of urgency or a desire to inform others [31]. Influencers, driven by the pursuit of attention and engagement, may amplify misinformation due to its sensational or emotionally resonant nature. In contrast, bots and fake accounts are typically programmed to deliberately spread false information with the intent to create confusion and panic [21,27]. These actors contribute to a feedback loop, whereby misinformation gains visibility and traction, becoming increasingly difficult to correct once it has spread widely [32].

Additionally, the design of social media platforms, which prioritizes immediacy and engagement, plays a pivotal role in the amplification of misinformation. Content such as exaggerated reports of disaster severity, false safety procedures, or fabricated health advisories often spreads more rapidly than verified information from official sources [28,32,33].

Algorithms that prioritize engaging content over factual accuracy contribute significantly to this phenomenon [5]. Zhai et al [9] demonstrated how social network dynamics and sentiment contagion fuel the spread of misinformation, particularly in emotionally charged disaster contexts. Rapid content sharing without verification contributes to what is referred to as a misinformation cascade, making it challenging for the public to distinguish between accurate and false information.

Users often place trust in their personal networks for disaster-related updates, assuming credibility based on social proximity rather than content accuracy. The misplaced trust exacerbates the problem. During Hurricane Sandy, for example, exaggerated reports of damage and doctored images circulated widely on social media, causing unnecessary fear and panic [4,8].

As misinformation spreads, it creates confusion and panic, directly undermining the effectiveness of official communication channels during disasters [4,6,8,9,12-15]. It misguides public behavior and erodes trust in official communications, thereby complicating disaster response efforts [6,13]. The convergence between misinformation origin and its public impact reveals the challenge of maintaining public trust and ensuring effective communication during disasters [8].

#### Public Impact

The societal and psychological effects of misinformation during disasters are profound. The misinformation distorts public perceptions, leading to confusion and inappropriate actions, such as unnecessary evacuations or ignoring official guidance [9]. This confusion, compounded by the public’s increasing reliance on social media for real-time updates, erodes trust in credible information and reduces compliance with official instructions [5,13].

The resulting behavior shifts, driven by misinformation, can delay coordinated disaster responses, misallocate resources, and undermine the overall effectiveness of emergency operations [6]. Dallo et al [13] similarly observed, stating that misinformation generates widespread confusion and fear among the public, significantly impeding disaster management efforts.

As reliance on social media increases, distinguishing between verified and false information becomes increasingly difficult, further diminishing public trust and adherence to official directives [5]. The erosion of public trust weakens effective communication and coordination during disaster response, contributing to fragmented and delayed decision-making [4-6,8,12-15].

Heightened emotional states during crises amplify the damaging effects of misinformation, destabilizing already fragile situations. For instance, during Hurricane Sandy and the 2018 floods in Kerala, misinformation fueled public confusion and anxiety, disrupting emergency responses and delaying critical relief efforts [6,8].

Moreover, beyond immediate operational challenges, the long-term mental health consequences of exposure to misinformation are significant. Anxiety, depression, and posttraumatic stress disorder may arise when receiving misinformation about the safety of affected areas or the availability of essential resources, placing further strain on mental health services [6,34,35].

The intersection between public impact and emergency response disruption becomes evident in the actions of misinformed individuals. Fueled by misinformation, public behaviors such as confusion, panic, and inappropriate actions directly undermine the efficacy of official communications and disaster response strategies [4,9,13]. These actions not only hinder response coordination but also lead to the misallocation of resources, ultimately reducing the effectiveness of emergency operations [6].

#### Emergency Response Disruption

While social media serves as a vital tool for crisis communication, it simultaneously poses significant challenges to emergency response operations. The rapid spread of misinformation can saturate communication channels, misdirect resources, and create false alerts, all of which complicate coordination and operational effectiveness [9,12,14].

Emergency responders are frequently forced to simultaneously manage both the actual disaster and counteract the spread of misinformation. This dual burden diverts attention and resources from where they are most urgently needed, thereby weakening the overall emergency response effort [6,9,12,14,15].

Disruption in resource deployment further complicates crisis management, particularly when misinformation about aid availability, safe zones, or evacuation procedures leads to confusion and misallocation. Events such as earthquakes and hurricanes have demonstrated how such misinformation delays relief efforts and overwhelms emergency services [6,9]. As responders are required to counter misinformation while managing real-time emergencies, operational delays become inevitable [6,8,9,12]. Compounding the problem is the speed at which misinformation spreads, often outpacing official corrections, making debunking efforts an ongoing challenge [4,5]. Hunt et al [5] observed that the effectiveness of debunking strategies is heavily dependent on both the timing and platform used. When corrective messaging lags, public safety is undermined and trust in emergency services erodes [9,14,15].

The interplay between misinformation origin, public impact, and emergency response disruption highlights the compounded challenges misinformation creates during disasters. As illustrated in Figure 2, the domain overlap reinforces a feedback loop showing how misinformation alters public behavior, amplifies panic, and disrupts emergency response operations, collectively degrading the effectiveness of crisis management and compromising public safety. Addressing these challenges requires robust mechanisms to combat them. Governments, social media platforms, and relevant organizations must collaborate to enhance public awareness, refine communication strategies, and build public resilience against misinformation. By understanding how misinformation spreads and impacts both the public and emergency services, more effective interventions can be developed to strengthen disaster resilience and improve public health outcomes.

## Discussion

### Principal Findings

The narrative review has critically evaluated the spread of misinformation on social media in the context of natural disasters, shedding light on key challenges, implications, and potential solutions.

A conceptual model was developed to illustrate the interconnected relationships between misinformation origin, public impact, and disaster response, grounded in evidence from the 9 reviewed studies.

The main findings reveal the dual role of social media during disasters; while facilitating the rapid dissemination of vital information, they also serve as a vector for misinformation. The speed and scale at which the misinformation spreads can undermine official communication efforts, create public confusion, disrupt disaster resilience, hinder public health efforts, and divert critical emergency resources [6,35]. Several studies reported cases in which misinformation regarding aid availability and safe locations led to unnecessary chaos, overwhelming emergency services, and delayed assistance to those in need [6,8,12,36]. When the public perceives official sources as unreliable or slow, they increasingly turn to unofficial and less credible alternatives, heightening the risk of acting on misinformation [28].

Additionally, the erosion of trust in official communications weakens community resilience [14] and has psychological implications that extend beyond the immediate disaster response. Exposure to misinformation has been associated with long-term mental health impacts, including anxiety, stress, and posttraumatic symptoms, especially when the public is misinformed about safety conditions or resource availability [4,37].

Addressing this duality requires a deep understanding of the origins of misinformation, its public impact, and its capacity to disrupt disaster response systems.

The 3 interconnected concepts—misinformation origin, public impact, and emergency response disruption—emerged across the studies and informed the development of the conceptual model (Figure 2). By illustrating the overlap between these domains, the model provides a structured framework for understanding how misinformation disrupts public health, emergency response effectiveness, and disaster resilience.

Misinformation during disasters originates from a variety of sources, including ordinary users, influencers, and bots [4,6,13-15]. While some share content with good intentions, others, particularly influencers, can unintentionally amplify false narratives due to their large followings [6,9,12,13,38]. Automated bots further exacerbate the problem by generating and spreading misinformation that appears legitimate through tactics such as hashtag hijacking and the use of official links, as observed during the 2013 Moore Tornado [8].

The rapid spread of misinformation is driven in part by the algorithmic architecture of social media platforms, which prioritizes engagement-based content (shares, likes, and comments) over accuracy [5]. This environment facilitates the viral dissemination of misinformation, contributing to heightened stress, confusion, anxiety, and public panic [5,35].

Numerous studies confirm the detrimental effects of misinformation on public well-being during disasters. When individuals receive conflicting reports, their trust in official sources diminishes, leading to heightened emotional responses and behavior that undermines response efforts, such as unnecessary evacuations or disregard for safety protocols [4,5,14,35]. Events like Hurricane Sandy and the Kerala floods exemplify how misinformation and increased public anxiety eroded trust in official communications and complicated relief operations [4,6,8].

The misallocation of resources is another consequence of misinformation. False alerts have repeatedly caused emergency services to divert attention to noncritical areas, delaying vital assistance elsewhere.

For instance, during Hurricane Sandy and the 2013 Moore Tornado, misinformation about resource availability misled responders and complicated coordination, while during the Kerala floods, false data led to misdirected relief operations [4,6,8,9,36]. These disruptions illustrate how misinformation not only undermines immediate disaster response efforts but also impairs longer-term coordination and recovery. The psychological burden faced by affected populations is further intensified when community recovery is delayed due to the diversion of resources and persistent misinformation [36].

From the evidence, several lessons emerge. First, the speed of the misinformation spread necessitates proactive and timely countermeasures. Once misinformation gains traction, it often outpaces correction efforts, causing widespread harm. Real-time debunking campaigns led by trusted authorities must be prioritized and well-resourced to counteract this effect [8,9,14]. Communication strategies must also emphasize accuracy, clarity, and empathy to rebuild public trust [6,14,39].

Second, public education is vital. Preemptive educational initiatives that promote digital literacy and critical thinking can empower individuals to evaluate online information and reject misinformation [8,14,40]. These campaigns should target all demographics, including vulnerable groups such as older adults who are more susceptible to false narratives [13]. By fostering media literacy, these campaigns can reduce the spread of misinformation and enhance public resilience [14,41].

Third, technological advances should be leveraged. AI detection tools for real-time verification can identify patterns and flag or remove misinformation before it spreads widely [12,42]. These technologies must be integrated into disaster communication systems to enhance responsiveness and limit harm.

Early intervention is critical. Rapid deployment of countermeasures can contain the spread of misinformation and reduce its impact. Delayed responses, by contrast, allow false narratives to proliferate, compounding the damage [8,9].

To conclude, future efforts must focus on a combination of proactive strategies, technological innovation, and public education to effectively combat the challenges posed by misinformation during disasters. Disaster preparedness plans must formally integrate management of misinformation as a key component, ensuring that emergency responders are equipped to swiftly identify and address false narratives.

The discussion should be interpreted in light of the narrative review’s strengths and limitations.

### Strengths

A notable strength of this narrative review lies in its robust and transparent methodological framework, which adheres to the established guidelines from the Joanna Briggs Institute and the Cochrane Handbook for Systematic Reviews. The rigorous observance of PRISMA guidelines further ensures methodological transparency, enhancing the reliability and reproducibility of the findings.

Additionally, this review synthesizes evidence from a diverse array of studies, incorporating data from different geographic regions and varied natural disaster contexts. By not imposing date restrictions, this paper captures a broad temporal perspective, allowing for an inclusive assessment of the evolving dynamics of misinformation on social media during natural disasters.

Furthermore, the conceptual model developed in this narrative review advances the literature by illustrating the complex interrelationships between misinformation origins, public impacts, and disruptions to emergency response systems.

#### Limitations

There may be several limitations of the narrative review.

- The retrieved results included only the studies that were indexed in PubMed or found in a free search on Google Scholar. Thus, any studies not indexed on these sites were excluded from the review.
- The selection of search terms might not have been sufficiently comprehensive to capture all existing literature on misinformation on social media during natural disasters.
- The review did not examine narrative, social, and other theories posed as reasons for the spread of the misinformation.
- Language restrictions to English-only studies may limit the generalizability of the findings.

#### Conclusions

This narrative review critically examined the role of misinformation on social media during disasters, specifically natural hazards, highlighting its challenges, implications, and potential solutions. The review highlights the pervasive and damaging impact of misinformation, including its disruption of disaster response, erosion of public trust, and amplification of psychological distress. The conceptual model developed (Figure 2) provides a structured framework to deepen understanding of these interconnected dynamics.

The findings also demonstrate the complex, dual role that social media plays during disasters. While it facilitates the rapid dissemination of vital information, it simultaneously acts as a breeding ground for misinformation. The speed and scale at which misinformation spreads can undermine official communications, erode public trust, and create confusion and anxiety. These disruptions not only compromise disaster resilience but also hinder the effectiveness of emergency response systems and degrade public health outcomes, as illustrated by the conceptual model.

Moving forward, proactive strategies, technological innovation, and public education must be prioritized. This includes integrating misinformation management into disaster preparedness plans, enhancing public awareness, deploying advanced verification tools, and fostering trust in credible sources. By combining these approaches, individuals and communities will be better equipped to navigate the challenges posed by misinformation, thereby strengthening disaster resilience and improving public health outcomes.

In addition, future research, currently limited in scope, should focus on the following areas.

- A clearer understanding of the current state of misinformation during natural disasters.
- The experiences and perspectives of public health and disaster response professionals, particularly how misinformation affects roles, decision-making, and the effectiveness of response strategy.
- Emotional and psychological dimensions of misinformation in the context of natural hazards.

Ongoing research and policy efforts are critical for refining disaster preparedness and ensuring that future disaster responses are not compromised by the harmful effects of misinformation.

## Supplementary material

10.2196/70413 Multimedia Appendix 1 Tables used for data extraction.

10.2196/70413 Multimedia Appendix 2 Type of natural disasters.

10.2196/70413 Multimedia Appendix 3 Type of misinformation spread.

10.2196/70413 Multimedia Appendix 4 Public impact because of exposure to misinformation extrapolated from the 9 papers.

## Abbreviations

AI: artificial intelligence
EUPHA: The European Public Health Association
HTA: Health Technology Assessment
MeSH: Medical Subject Headings
PRISMA: Preferred Reporting Items for Systematic Reviews and Meta-Analyses
PROSPERO: International Prospective Register of Systematic Reviews
R&D: Research and Development
