# Supplementary material for: The Impact of Misinformation on Social Media in the Context of Natural Disasters: Narrative Review
Source: JMIR Infodemiology. 2025 Jul 31;5:e70413. doi: 10.2196/70413 (PMC12313155; doi:10.2196/70413)
Supplement: Multimedia Appendix 1 [file infodemiology-v5-e70413-s001.docx]

**Table S1. Data extraction (I).**

| **Author and study** | **Country of origin** | **Study type and social media platform/s** | **Study size** | **Purpose** | **Who is communicating** |
| --- | --- | --- | --- | --- | --- |
| Zhai et al [9] | USA | Case study and social network Analysis / Twitter (X). | 691 misinformation-sharing users. | Decipher the spread of disaster misinformation and its correction through the case study of the disaster rumour during Hurricane Sandy (2012) on Twitter. | General public |
| Dallo et al [13] | Global | Content analysis / Twitter (X)r | 82,129 tweets | Assess the dynamics and frequencies of earthquake prediction misinformation and information tweets, characteristic differences between - earthquake prediction misinformation tweets and information tweets - misinformation and users who tweet accurate earthquake notifications, and users who actively counter earthquake misinformation - earthquake prediction misinformation and not-misinformation tweets regarding the usage of media and URLs. | general public |
| Vasudevan and Alathur [6] | India | Case study / social media | 561  surveys who were affected by the flood in Kerala. | Determine the impact of social media and misinformation during distress (Heavy Flooding). | general public |
| Rajdev and Lee [8] | USA | Thesis: Case study / Twitter (X). | 1% sample tweets posted during a period of each of the two events. | Selected two well-known disasters, Moore Tornado and Hurricane Sandy and analysed labelled datasets for patterns in messages. | Users on social media, differentiated by legitimate, spam, and fake accounts. |
| Hunt et al [5] | USA | Case study / Twitter (X) | Hurricane Harvey: 2,032 unique tweets (1,440 debunking tweets).  Hurricane Irma: 601 unique tweets (259 debunking tweets). | To analyse the lifespan of false immigration rumours during Hurricanes Harvey and Irma.  To understand the effectiveness of debunking efforts on Twitter. | Government organisations,  News agencies,  NGOs and  Verified and unverified Twitter users. |
| Oh et al [14] | Haiti | Content analysis / Twitter | 962 tweets | To explore the dynamics of rumour and misinformation during extreme events using Twitter data from the Haiti Earthquake and understand how anxiety and informational uncertainty influence rumour spread. | Twitter users from around the world, including eyewitnesses in Haiti and those participating in the global discussion. |
| King and Wang [12] | USA | Big data-driven analysis of real versus misinformation using Twitter data. | 42 million tweets with 3,589 original verified real or false tweets cross-checked with fact-checking websites and relevant federal agencies. | To investigate the factors affecting the virality of real news versus misinformation on social media during a crisis event, with a focus on time-based, content-based, and user-based factors. | Twitter users, including regular users and potential automated entities (bots), during the crisis of Hurricane Harvey. |
| Gupta et al [4] | USA | Content analysis/ Twitter | 1.8 million tweets from 1.2 million unique users, with 10,350 tweets containing fake images and 5,767 tweets containing real images. | To characterise the spread of fake images on Twitter during Hurricane Sandy and to identify methods for detecting fake images using automated techniques. | Twitter users, including both general users and malicious entities who spread fake images. |
| Abdullah et al [15] | Japan | Survey / Twitter | 133 participants (students from Iwate Prefectural University, Japan). | To investigate user actions and decision-making when retweeting messages on Twitter during disasters and to understand how to reduce the spread of misinformation. | Twitter users (students in this study). |

**Table S2. Data extraction (II).**

| **Author** | **Type of disaster and misinformation** | **Misinformation identified** | **Impact identified** | **Who is impacted** |
| --- | --- | --- | --- | --- |
| Zhai et al [9] | Hurricane / Disaster rumour misinformation during Hurricane Sandy (2012). | Rumours spread on Twitter during Hurricane Sandy, including false information about the disaster's impact and situation, such as exaggerated damages or incorrect emergency instructions. | This misinformation may have caused confusion, led to improper responses to the disaster, and possibly diverted resources from where they were needed. Moreover, it could have contributed to public fear and stress during an already challenging situation. | Individuals directly affected by Hurricane Sandy, emergency responders, public safety officials, the general public following the disaster on Twitter, and Twitter users involved in disseminating and correcting information. |
| Dallo et al [13] | Earthquake / earthquake prediction misinformation. | Misleading tweets about the ability to predict earthquakes, sometimes tagging or linking official earthquake notifications from credible sources to give the false impression of legitimacy. | Misinformation about earthquake predictions can lead to unnecessary panic, undermine public trust in actual scientific information, and distract from genuine preparedness efforts. | General public on Twitter, especially those in earthquake-prone areas, official institutions like earthquake notification centres, and scientists who may have their credible information undercut by false predictions. |
| Vasudevan and Alathur [6] | heavy floods / Impulsive misinformation during distress | Unverified and hastily shared information on social media in the context of a natural calamity, leading to disruptions in relief operations. | Misinformation has caused interruptions in relief and rescue operations, potentially endangering lives and resources. | The affected people in the disaster-stricken area, individuals engaged in relief and rescue operations, and the broader community of social media users coordinating aid. |
| Rajdev and Lee [8]. | 2013 Moore Tornado, Hurricane Sandy. | Presence of URLs linked to malware or misinformation, use of hashtags. | Legitimate vs. non-legitimate content, spam, and fake messages distinguished by URLs and other features not able to be distinguished by the general public. | General public, in the affected areas. |
| Hunt et al [5] | Hurricanes Harvey and Irma.  Misinformation is about immigration enforcement at evacuation sites and shelters. | False claims that immigration status checks were being conducted at evacuation sites and shelters. | Prolonged spread of false information can lead to unnecessary panic and deter affected individuals from seeking help. | General public, especially immigrant communities potentially fearful of seeking aid. |
| Oh et al [14] | Haiti Earthquake, 2010 / False claims about aid offers, such as rumours about UPS shipping packages to Haiti for free or airlines taking medical personnel for free. | Rumours were prevalent, including claims about free shipments to Haiti by UPS and free flights for medical personnel by airlines, which were later debunked. | The spread of misinformation contributed to confusion and false hope but was mitigated by the dissemination of credible information from reliable sources like media outlets and organisations. | Primarily the people in Haiti and the global community engaging with the disaster response efforts through social media. |
| King and Wang [12] | Hurricane Harvey/ Tweets containing false information related to the crisis, such as exaggerated claims or incorrect details about the situation and rescue efforts. | The study identified that misinformation tends to spread faster than real news, especially when tweets are novel, contain negative sentiment, or have low readability. | Misinformation spreads more virally than real news, potentially misleading large audiences during the crisis and affecting response and perception of the disaster. | Twitter users, crisis responders, and the general public who rely on social media for information during the crisis. |
| Gupta et al [4]. | Hurricane Sandy / Fake images related to the storm, such as sharks in flooded streets and exaggerated storm scenes. | Specific examples include fake images of sharks swimming in the streets and manipulated photos of storm damage. | The spread of fake images caused panic and chaos among people during the crisis, with a significant viral spread of misinformation through retweets. | The general public affected by Hurricane Sandy, particularly those relying on social media for real-time information. |
| Abdullah et al [15] | Generically focused on retweets during disasters, including unverified information and rumours. | The study highlights the issue of users retweeting information they consider important, without verifying its accuracy, leading to the spread of false or misleading information. | Spread of misinformation during disasters can lead to public confusion, delayed response efforts, and unnecessary panic. | The general public, disaster response teams, and those relying on social media for information during emergencies. |

**References**

4. Gupta A, Lamba H, Kumaraguru P, Joshi A. Faking Sandy: characterizing and identifying fake images on Twitter during Hurricane Sandy. Presented at: Proceedings of the 22nd International Conference on World Wide Web (WWW ’13 Companion); May 13-17, 2013:729-736; Rio de Janeiro Brazil. [doi: 10.1145/2487788.2488033]

5. Hunt K, Wang B, Zhuang J. Misinformation debunking and cross-platform information sharing through Twitter during Hurricanes Harvey and Irma: a case study on shelters and ID checks. Nat Hazards. Aug 2020;103(1):861-883. [doi: 10.1007/s11069-020-04016-6]

6. Vasudevan J, Alathur S. Misinformation in social media during disasters. Int J Inf Syst Soc Change. Jan 2022;13(1):1-15. URL: <https://services.igi-global.com/resolvedoi/resolve.aspx?doi=10.4018/IJISSC.20220101> [doi: 10.4018/IJISSC.303596]

8. United Nations Office for Disaster Risk Reduction (UNDRR). The Sendai Framework Terminology on Disaster Risk Reduction “Disaster.” 2017. URL: <https://www.undrr.org/terminology/disaster> [Accessed 2025-03-20]

9. Zhai W, Yu H, Song CY. Disaster misinformation and its corrections on social media: spatiotemporal proximity, social network, and sentiment contagion. Ann Am Assoc Geogr. Feb 7, 2024;114(2):408-435. [doi: 10.1080/24694452.2023.2271549]

12. King KK, Wang B. Diffusion of real versus misinformation during a crisis event: a big data-driven approach. Int J Inf Manage. Aug 2023;71:102390. [doi: 10.1016/j.ijinfomgt.2021.102390]

13. Dallo I, Elroy O, Fallou L, Komendantova N, Yosipof A. Dynamics and characteristics of misinformation related to earthquake predictions on Twitter. Sci Rep. Aug 17, 2023;13(1):13391. [doi: 10.1038/s41598-023-40399-9] [Medline: 37592002]

14. Oh O, Kwon KH, Rao HR. An exploration of social media in extreme events: rumor theory and twitter during the haiti earthquake 2010. Presented at: ICIS 2010 Proceedings. 231; 2010.URL: <https://aisel.aisnet.org/icis2010_submissions/231> [Accessed 2024-04-14]

15. Abdullah NA, Nishioka D, Tanaka Y, Murayama Y. User's action and decision making of retweet messages towards reducing misinformation spread during disaster. J Inf Process. 2015;23(1):31-40. [doi: 10.2197/ipsjjip.23.31]
