# Supplementary figures and images for: The Impact of Misinformation on Social Media in the Context of Natural Disasters: Narrative Review
Source: JMIR Infodemiology. 2025 Jul 31;5:e70413. doi: 10.2196/70413 (PMC12313155; doi:10.2196/70413)

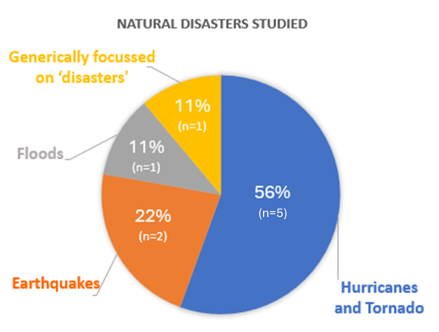

Supplement: Multimedia Appendix 2 [file infodemiology-v5-e70413-s002.png]

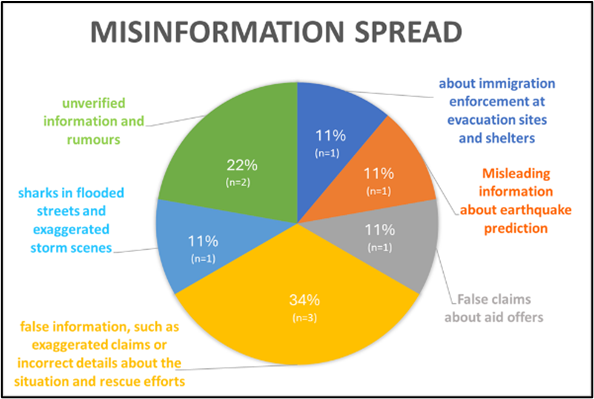

Supplement: Multimedia Appendix 3 [file infodemiology-v5-e70413-s003.png]

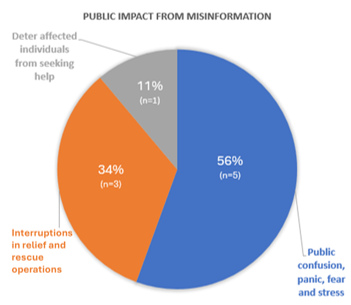

Supplement: Multimedia Appendix 4 [file infodemiology-v5-e70413-s004.png]
